# Fructosamine-3-Kinase as a Potential Treatment Option for Age-Related Macular Degeneration

**DOI:** 10.3390/jcm9092869

**Published:** 2020-09-04

**Authors:** Sander De Bruyne, Caroline Van den Broecke, Henk Vrielinck, Samira Khelifi, Olivier De Wever, Ken Bracke, Manon Huizing, Nezahat Boston, Jonas Himpe, Marijn Speeckaert, Anne Vral, Jo Van Dorpe, Elisabeth Van Aken, Joris R. Delanghe

**Affiliations:** 1 Department of Diagnostic Sciences, Ghent University, 9000 Ghent, Belgium; sanderR.debruyne@ugent.be (S.D.B.); jonas.himpe@ugent.be (J.H.); jo.vandorpe@ugent.be (J.V.D.); 2 Department of Pathology, Ghent University Hospital, 9000 Ghent, Belgium; caroline.vandenbroecke@azstlucas.be; 3 Department of Solid State Sciences, Ghent University, 9000 Ghent, Belgium; henk.vrielinck@ugent.be (H.V.); samira.khelifi@ugent.be (S.K.); 4 Department of Human Structure and Repair, Ghent University, 9000 Ghent, Belgium; olivier.dewever@ugent.be (O.D.W.); anne.vral@ugent.be (A.V.); 5 Department of Internal Medicine and Pediatrics, Ghent University, 9000 Ghent, Belgium; ken.bracke@ugent.be (K.B.); marijn.speeckaert@ugent.be (M.S.); 6 Biobank, Antwerp University Hospital, 2650 Antwerp, Belgium; manon.huizing@uza.be (M.H.); nezahat.boston@uza.be (N.B.); 7 Research Foundation Flanders, 1000 Brussels, Belgium; 8 Department of Head and Skin, Ghent University, 9000 Ghent, Belgium

**Keywords:** age-related macular degeneration, eye diseases, fructosamine-3-kinase, glycation end products, advanced, retinal drusen, therapeutics

## Abstract

Age-related macular degeneration is the leading cause of blindness in the developed world. Since advanced glycation end products (AGEs) are implicated in the pathogenesis of AMD through various lines of evidence, we investigated the potential of fructosamine-3-kinase (FN3K) in the disruption of retinal AGEs, drusenoid material and drusenoid lesions in patients with AMD. AGE-type autofluorescence was measured to evaluate the effects of FN3K on glycolaldehyde-induced AGE-modified neural porcine retinas and unmodified human neural retinas. Eye pairs from cigarette-smoke- and air-exposed mice were treated and evaluated histologically. Automated optical image analysis of human tissue sections was performed to compare control- and FN3K-treated drusen and near-infrared (NIR) microspectroscopy was performed to examine biochemical differences. Optical coherence tomography (OCT) was used to evaluate the effect of FN3K on drusenoid deposits after treatment of post-mortem human eyes. FN3K treatment provoked a significant decrease (41%) of AGE-related autofluorescence in the AGE-modified porcine retinas. Furthermore, treatment of human neural retinas resulted in significant decreases of autofluorescence (−24%). FN3K-treated murine eyes showed less drusenoid material. Pairwise comparison of drusen on tissue sections revealed significant changes in color intensity after FN3K treatment. NIR microspectroscopy uncovered clear spectral differences in drusenoid material (Bruch’s membrane) and drusen after FN3K treatment. Ex vivo treatment strongly reduced size of subretinal drusenoid lesions on OCT imaging (up to 83%). In conclusion, our study demonstrated for the first time a potential role of FN3K in the disruption of AGE-related retinal autofluorescence, drusenoid material and drusenoid lesions in patients with AMD.

## 1. Introduction

Age-related macular degeneration (AMD) is a degenerative disorder of the macular region of the retina that is associated with a progressive loss of central vision [1]. It is the leading cause of visual loss and blindness among older patients in industrialized countries [2], leading to a major impact on functional independence and quality of life with substantial socioeconomic implications [1,3]. Clinically, AMD can be classified as early-stage (medium-sized drusen and retinal pigmentary changes) to late stage [1]. Late-stage AMD can be neovascular (wet or exudative form) or non-neovascular (dry, atrophic or non-exudative form) [1]. While implementation of the recently developed anti-vascular endothelial growth factor therapeutic agents delays vision loss in over 90% of patients with wet AMD (which constitutes only 10% of the AMD patients) [2], currently there is no proven therapy for dry AMD, the most prevalent form [1,3,4]. Remaining untreated, dry AMD patients are at risk for substantial vision loss and progression to wet AMD [4].

While AMD is a complex multifactorial disorder with known dysregulations in complement, lipid, angiogenic, inflammatory and extracellular matrix pathways [1], advanced glycation end products (AGEs) are receiving considerable recognition as an important risk factor in its pathogenesis. Increased levels of AGEs have been found in the Bruch’s membrane, retinal pigment epithelium (RPE) and drusen of patients with AMD [5,6,7,8]. Formation of these protein-adducts is linked to reactions with glucose, lipid peroxidation and various α-oxaloaldehydes such as glycolaldehyde [8,9]. AGEs are regarded as significant abettors of age-related diseases by causing structural and functional impairment of proteins, which eventually results in neurodegeneration, irreversible changes in the extracellular matrix, pro-inflammatory signaling and vascular dysfunction [1,8,9].

Based on these findings, we can hypothesize that novel agents, which are able to prevent or reverse AGE formation, could offer great therapeutic potential. While fructosamine-3-kinase (FN3K) is known as an enzyme involved in natural cellular repair mechanisms to control non-enzymatic glycation of proteins [10,11], its potential in the disruption of retinal AGEs has never been investigated. The enzyme is more active in tissues with a long half-life (e.g., brain, erythrocytes and lens), but the expression of the genes for FN3K appears to be constitutive and unaffected by environmental signals [12]. We studied the potential of recombinant FN3K treatment in the disruption of AGE-related retinal autofluorescence (AF), assessed its effects after treatment of tissue sections originating from AMD patients and evaluated its effects on drusenoid material and lesions after intravitreal injection of post-mortem murine and human eyes.

## 2. Experimental Section

### 2.1. Recombinant Production of Fructosamine-3-Kinase

A gene coding for human FN3K (GenBank accession no. NP_071441.1) was codon-optimized for *Pichia pastoris* expression (SEQ ID N° 1) and cloned into the pKai61 *P. pastoris* expression vector [13]. The encoded gene contains an N-terminal His6-tag (MHHHHHH) in frame with a caspase-3 cleavage site (DEVD), and the expression is under control of the methanol inducible aldehyde oxidase 1 (AOX1) promoter. The vectors were linearized in the AOX1 promoter before transformation to *P. pastoris* (strain NRRL Y-11430) to promote homologous recombination in the endogenous AOX1 locus for stable integration into the genome. Stable integrants were cultured shaking at 28 °C in BMY buffered complex medium (100-mM potassium phosphate pH 6, 2% peptone, 1% yeast extract and 1% yeast nitrogen base without amino acids) complemented with 1% glycerol. After 48 h of growth, recombinant expression was induced by transfer to BMY medium complemented with 1% methanol. After 48 h of expression, cultures were centrifuged, supernatant was discarded and pellets were flash frozen in liquid nitrogen and stored at −20 °C. Pellets were thawed and resuspended in isolation buffer for protein extraction (50 mM sodium phosphate pH 8.0, 400 mM NaCl, 20 mmol/L imidazole, 100 mg/L reduced glutathion, 0.01 mM n-dodecyl β-D-maltoside and 1 mmol/L DTT). *Pichia pastoris* cells were mechanically disrupted. The cleared supernatant was purified by Ni^2+^ affinity chromatography, followed by gel filtration on a SuperDex 75 column (GE Healthcare). The protein eluted in FN3K sample buffer (20 mM Tris-HCl pH 8.0, 150 mM NaCl, 1 mM DTT) was identified as human FN3K by SDS-PAGE and Western blotting. Enzymatic activity was confirmed in a 1-deoxy-1-morpholino-D-fructose substrate-based assay. FN3K aliquots were flash frozen in liquid nitrogen and stored at −80 °C [14,15,16].

### 2.2. Autofluorescence Measurement of AGEs

Since fluorescence spectroscopy is a valuable and commonly employed method for the detection and measurement of autofluorescent AGEs [17,18], Maillard-type AF measurements (excitation 370 nm, emission 390–700 nm) were performed using a Flame miniature spectrometer (FLAME-S-VIS-NIR-ES, 350–1000 nm, Ocean Optics, Dunedin, FL, USA) equipped with a high-power LED light source (365 nm, Ocean Optics) and reflection probe (QR400-7-VIS-BX, Ocean Optics). Measurements were averaged over 128 scans. AF-values were calculated by dividing the average light intensity emitted per nm for the 407–677 nm range by the average light intensity per nm over the 342–407 nm range.

### 2.3. FN3K Treatment of AGE-Modified Neural Porcine Retinas

Porcine eyes (*n* = 20) were obtained from a local abattoir and stored at 4 °C until processing. Neural retinas were isolated through dissection by a trained ophthalmologist within 12 h post-mortem, transferred to a sterile 6-well plate (Thermo scientific, Roskilde, Denmark) and frozen at −20 °C. A retinal fragment was cut from each frozen retina and added to a well of a black 96-well plate for fluorescence measurements (FluoroNunc PolySorp, Thermo Fisher Scientific, Waltham, MA, USA). Subsequently, fluorescence measurements were performed at baseline for each retinal fragment at a fixed distance and 90° angle.

Since glycolaldehyde is a well-known component to modify proteins by AGE formation and has a documented role in the pathogenesis of AMD [8,9], AGE modification was performed by incubation of the retinal fragments with 200 µL of 25-mM glycolaldehyde dimer (crystalline form, Sigma-Aldrich) in phosphate buffered saline (PBS) for 3 h at 37 °C. After incubation, the active agents were carefully washed away, and retinal fragments were stored overnight (4 °C) until termination of the chemical reaction was completed.

Finally, in vitro deglycation was initiated using a solution containing 125 µg/mL FN3K and a fixed amount of adenosine triphosphate (ATP, 12.5 mM, Sigma-Aldrich) and magnesiumdichloride (MgCl_2_, 5 mM, Sigma-Aldrich) in PBS. Twenty microliters of the final FN3K solution were added to each retinal fragment and incubated for 3 h at 37 °C. Fluorescence measurements were performed immediately after the addition of the FN3K solution and repeated after the incubation period. As a control experiment, 5 retinal fragments were processed in a similar way. However, the fragments were control treated with 20 µL of PBS after AGE-modification.

### 2.4. FN3K Treatment of Human Neural Retinas

Four human neural retinas were isolated through dissection by a trained ophthalmologist within 12 h post-mortem and immediately transferred to a sterile 6-well plate and stored at 4 °C in RPMI-1640 medium (Sigma-Aldrich, St. Louis, MO, USA). One donor eye was obtained from a phakic 79-year-old patient with stage 1 AMD (Age-Related Eye Disease Study Research Group (AREDS) staging [19]), polymyalgia rheumatica, heart failure and severe aortic valve failure. Two other donor eyes were prevailed from a phakic 87-year-old patient with stage 1 AMD, cardiac failure and lung edema. In addition, one donor eye was collected from a 79-year-old male with stage 2 AMD, multiple myeloma, benign prostatic hyperplasia and arrhythmia (right eye; the left eye was used for the ex vivo experiment described in Section 2.8). The experiment was started within 48 h by removing the RPMI medium and carefully washing the retinas with PBS. Subsequently, fluorescence measurements were performed at baseline on each retina at 30 different retinal locations with a fixed distance and 90° angle. Afterwards, in vitro deglycation was initiated using a solution containing 1.6 µg/mL FN3K and a fixed amount of ATP (2.5 mM) and MgCl_2_ (1 mM) in PBS. Two milliliters of the final FN3K solution were added to each retina well and incubated for 24 h at 37 °C. After the treatment procedure, all wells were washed with PBS and fluorescence measurements were performed again.

### 2.5. FN3K Treatment of Murine Eyes

Since cigarette smoke is an important risk factor for AMD [20], eye pairs were enucleated from one C57BL/6 wild-type mouse exposed to air and one C57BL/6 wild-type mouse exposed to cigarette smoke for 6 months. While each left eye was control-treated with an intravitreal injection composed of 5 µL of a solution containing 2.5 mM ATP and 1 mM MgCl_2_ in PBS, each right eye was treated with an intravitreal injection containing 5 µL of the FN3K solution described in Section 2.4. Eyes were fixed in 10% neutral-buffered formalin for 6–24 h. After fixation, samples were routinely processed using a Tissue-Tek^®^ VIP^®^ (Sakura, Torrance, CA, USA), embedded in paraffin, and 1.5-μm tissue sections were prepared, stained with hematoxylin and eosin (HE) and finally cover-slipped. Blinded evaluations of the tissue sections were performed by an expert pathologist through standard histological examination.

### 2.6. FN3K Treatment of Human Eye Tissue Sections Containing Drusen

Donor eyes were obtained from 2 patients with stage 3 AMD (age > 70 years). Enucleated eyes were largely processed in a similar way to the murine eyes. However, after tissue sectioning, samples were deparaffinized prior to treatment by consecutive submerging in xylene (3 × 1.5 min), alcohol (90% 2 × 1 min; 75% 1 × 1 min) and rinsing in water. Afterwards, the slides were dried for 10 min at 60 °C. For control treatment, one section was covered with 1 mL of the ATP/MgCl_2_ solution described in Section 2.4. For FN3K treatment, an adjacent section was treated using 1 mL of the FN3K solution described in Section 2.4. Both sections were incubated at 37 °C for 24 h. After incubation, tissue sections were carefully washed with distilled water and dried overnight at 37 °C. Sections were then stained, cover-slipped and evaluated as described above. In addition, HE stained tissue sections were scanned by the Olympus dotSlide Digital Virtual Microscopy System and processed using the OlyVIA viewer program (Olympus Corporation, Tokyo, Japan). For subsequent image analysis, ImageJ v1.8.0 (NIH, http://rsb.info.nih.gov/ij) was used. Red (R), green (G) and blue (B) intensity values were calculated using the RGB Measure plug-in.

### 2.7. Near-Infrared Microspectroscopy and Multivariate Data Analysis

Infrared (IR) microspectroscopy combines light microscopy with IR spectroscopy and is a powerful analytical technique to obtain biochemically selective visualizations of tissue sections [21,22]. IR spectroscopy is based on the principle that different regions of IR light are absorbed by various molecules within tissues (e.g., carbohydrates, proteins and lipids) [22,23]. In a typical IR microspectroscopy system, visible light is employed to visualize and target areas of interest on tissue sections. Once that particular region is found (e.g., a specific drusen), the system switches to the IR configuration and IR light is beamed onto the predefined target [22]. To obtain a chemical fingerprint of the drusenoid material (Bruch’s membrane) and drusen lesions on the same tissue sections used for light microscopic examination, Fourier transform near-infrared (FT-NIR) transmission microspectra were recorded with a Bruker Hyperion 2000 microscope coupled to a Bruker Vertex 80v FTIR spectrometer (Bruker, MA, USA) operating with a halogen light source, a CaF_2_ beam splitter and an InGaAs detector. The objective magnification of the microscope was set at 15× and the aperture at 20 µm × 20 µm. The background was collected with 800 co-adds. Spectra were recorded at a resolution of 16 cm^−1^ in the range from 12,000 to 4000 cm^−1^ (800 scans). Spectral data analysis was performed using SIMCA software version 15.0 (MKS Data Analytics Solutions, Malmö, Sweden). Different preprocessing steps were performed to minimize irrelevant light scatter and standardize the spectroscopic signals. Differentiation was performed to accentuate small structural differences and reduce baseline effects [24]. Standard normal variate normalization (SNV) was performed to eliminate multiplicative scaling effects and additive baseline offset variations [24]. After preprocessing, spectral data were analyzed by unsupervised pattern recognition methods, such as principal component analysis (PCA), and supervised pattern recognition methods, such as partial least squares-discriminant analysis (PLS-DA). PLS-DA is a useful method to illustrate which variables are responsible for discrimination between 2 distinct groups.

### 2.8. Ex Vivo Intravitreal FN3K Treatment of Post-Mortem Human Eyes

**Figure 1:**
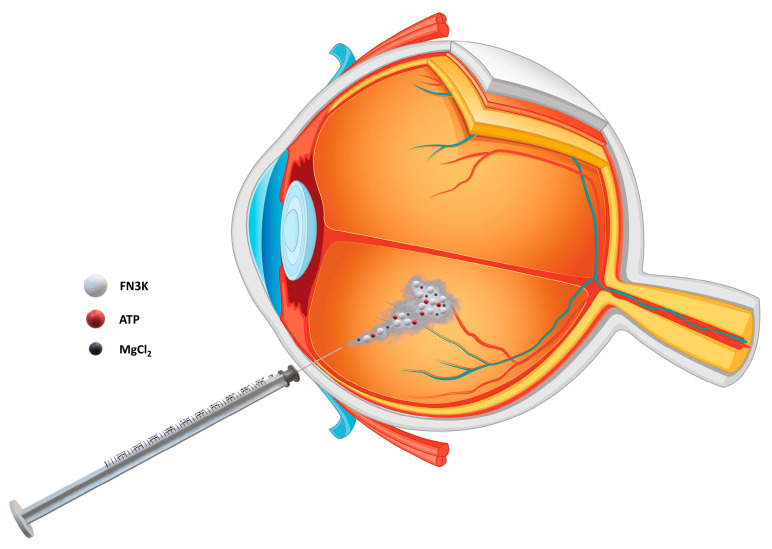
Intravitreal eye injection of a solution containing fructosamine-3-kinase (FN3K) and its cofactors adenosine triphosphate (ATP) and magnesiumdichloride (MgCl_2_).

Post-mortem eyes obtained from the Biobank Antwerp (Antwerp, Belgium, ID: BE 71030031000) were eviscerated from fresh cadavers, transported on ice and evaluated for the presence of drusen using optical coherence tomography (OCT). Eyes were prevailed from a 92-year-old donor with stage 3 AMD, phakic eyes and cardiac failure (right eye); a 74-year-old donor with an extensive ophthalmological history of stage 4 AMD, repair of retinal detachment, cataract and non-proliferative diabetic retinopathy (right eye); and the contralateral left eye of the 79-year-old donor with stage 2 AMD described in Section 2.4. For imaging of subretinal drusenoid lesions, post-mortem eyes were positioned in front of the camera and blue-reflectance images (wavelength 488 nm, scan size 512 pixels, 6 mm length scans and 20° angle) were captured with HRA + OCT Spectralis (Heidelberg Engineering, Germany). To prove the ability of FN3K to disrupt drusenoid material after intravitreal injection, whole eyes containing subretinal drusenoid lesions were treated as follows: eyes were first warmed up to 37 °C for 30 min, then positioned and immobilized at the OCT machine and intravitreally injected with 50 µL of the FN3K solution described in Section 2.4 (Figure 1). The formulation of the solution was improved by adding thiosulfate (0.1 M) and hyaluronidase (5 U/mL). While thiosulfate was used to reduce calcified structures in drusen which have the potential to act as shields impairing adequate penetration of the enzyme [25,26], hyaluronidase (a well-known adjunct to local anesthetics in ophthalmic surgery) was chosen as an agent to reduce viscosity of the vitreous humor [27,28,29]. In the in vivo situation, saccadic eye movements induce a flow in the vitreous humor of the eye, which positively influences the dispersion of drugs injected into the vitreous chamber [30]. However, since those rotations are absent in post-mortem eyes, the viscous nature of the vitreous fluid could otherwise impair adequate distribution of the enzyme [27]. Effect of treatment on OCT imaging was evaluated 2 h after injection. No clear distinction was noticed between different layers of the inner and outer retina due to impaired visualization through severely decompensated cornea, as eyes were rejected for corneal transplantation. While removal of the anterior segment (cornea and lens) could allow more precise visualization of AMD features (e.g., Utah protocol [31]), treating a flat retina with FN3K would not be representative for the in vivo situation. The area (µm^2^) of subretinal drusenoid deposits was calculated using ImageJ. The study was approved by the local ethics committee (Belgian registration number B670201838497). 

### 2.9. Statistical Analysis

Statistical analysis was performed using GraphPad Prism version 8.4.3. Normality of the data was assessed by the Shapiro–Wilk test. Normally distributed data are presented as mean ± standard deviation (SD), non-normally distributed data as median with the interquartile range (IQR). For non-normally distributed data, unpaired differences between 2 groups were assessed using the Mann–Whitney U test. For normally distributed data, pairwise comparisons between 2 and 3 groups were accomplished with paired *t* tests and repeated measures one-way analysis of variance (ANOVA), respectively. A *P* value < 0.05 was considered a priori to be statistically significant.

## 3. Results

### 3.1. FN3K Treatment of AGE-Modified Neural Porcine Retinas and Human Neural Retinas Reduces AGE-Related Autofluorescence

**Figure 2:**
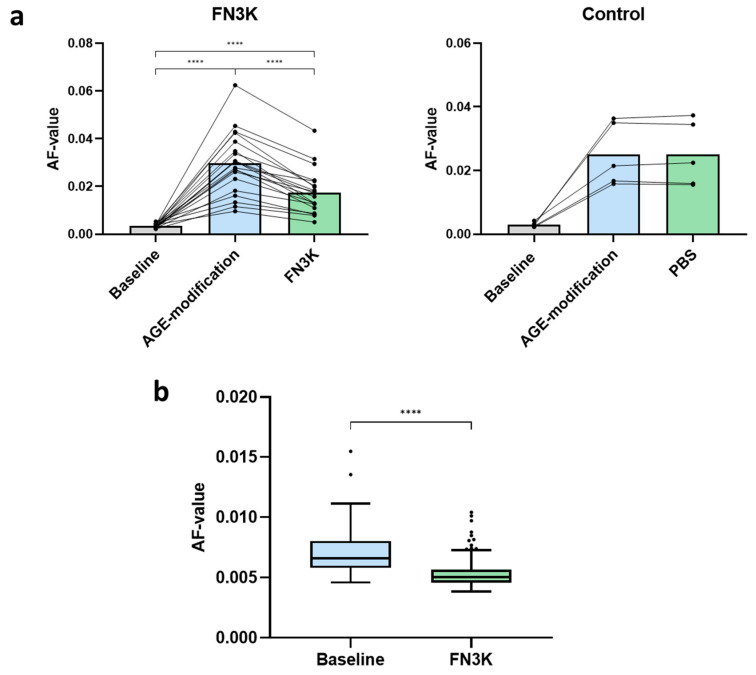
(**a**) Autofluorescence (AF) values of glycolaldehyde-induced advanced glycation end product (AGE)-modified porcine neural retinas after control (*n* = 5) and fructosamine-3-kinase (FN3K, *n* = 20) treatment. The bars represent the mean. PBS = phosphate buffered saline. (**b**) Box-and-whisker plot illustrating AF-values at 30 different retinal locations of four different post-mortem human neural retinas from stage 1 (*n* = 3) and stage 2 (*n* = 1) AMD patients at baseline (*n* = 120) and after treatment with FN3K (*n* = 120). **** *P* < 0.0001.

Figure 2a shows the AF-values of neural porcine retinas (*n* = 20) at baseline, after AGE-modification and finally after FN3K treatment of the AGE-modified retinas. AGE-specific AF-values significantly increased in the retinal fragments after incubation with 25-mM glycolaldehyde (AF-value 0.030 ± 0.0092) compared to baseline levels (AF-value 0.0035 ± 0.00083, *P* < 0.0001). Subsequently, FN3K treatment provoked a significant decrease of AF-values (AF-value 0.018 ± 0.0092, −41%, *P* < 0.0001). No changes were found after control treatment of AGE-modified retinal fragments (*n* = 5). As mentioned in the Experimental Section 2.4, FN3K treatment was also performed on four post-mortem human neural retinas obtained from three AMD patients. Baseline measurements and measurements after FN3K treatment were performed in 30 different retinal regions for each retina. For all measurements, a significant decrease (24%) of median AF-values was noted when comparing levels at baseline (*n* = 120, median 0.0066, IQR 0.0058–0.0080) and after FN3K (*n* = 120, median 0.0050, IQR 0.0046–0.0057) treatment (*P* < 0.0001, Figure 2b).

### 3.2. Murine Eyes Treated Intravitreally with FN3K Show Less Drusenoid Material and Lesions on Stained Tissue Sections

**Figure 3:**
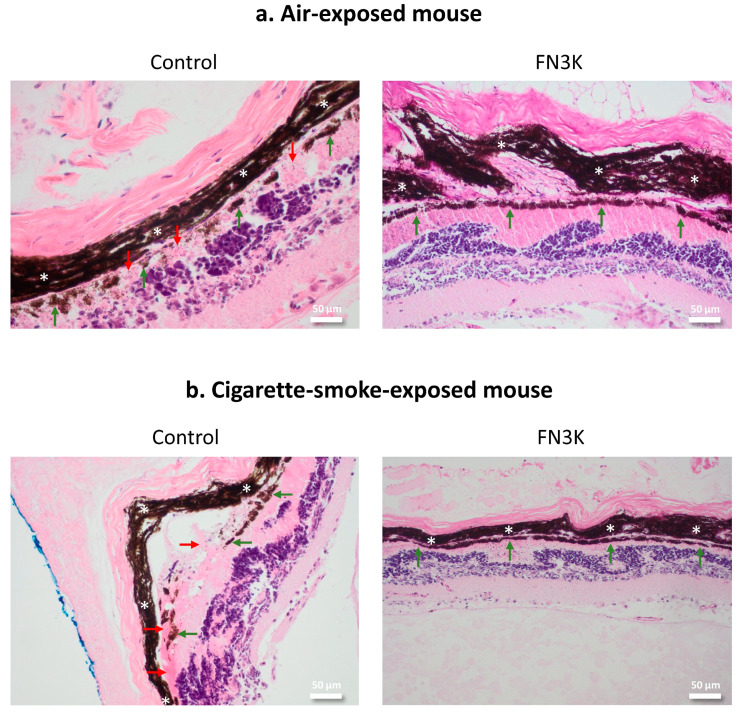
Hematoxylin and eosin stained tissue sections of eyes from one non-smoking mouse (**a**) and one mouse exposed to cigarette smoke (**b**) after intravitreal injection of control or FN3K treatment (magnification 20×). The choroid is indicated with white asterisks, drusenoid material with red arrows and the retinal pigment epithelium with green arrows. The scale bars are 50 µm.

Figure 3 shows HE stained sections of the non-smoking (Figure 3a) and smoking mouse (Figure 3b) after control and FN3K treatment. The neuroretina showed no pathological differences between the control- and FN3K-treated sections. The folding of the retina is an artefact occurring in some eyes after tissue processing (i.e., fixation, embedding and angle of cutting of the specimen). It can be ascribed to differences in the structure of the eye layers. The fact that several layers (i.e., sclera, choroid and neuroretina) show the same folding pattern indicates that it is an artifact rather than a pathological event. Control-treated eyes showed the presence of amorphous eosinophilic material between the RPE and Bruch’s membrane, concordant with drusen. The staining intensity and size of the lesions were variable. While the control-treated non-smoking mouse eye showed several small lesions with irregular staining of the RPE and decreased pigment intensity above and surrounding the drusen, the control-treated smoking mouse eye showed a larger lesion with a discontinuous aspect of the RPE. For both control-treated eyes, the RPE was in most cases less intense and more irregular in areas covering the drusen, whereas, in their FN3K-treated counterparts, the RPE seemed to be more regular and drusenoid lesions were less evident. Inflammatory changes were not observed.

### 3.3. FN3K Treatment Reduces the Color Intensity and Area of Drusenoid Lesions on Stained Human Tissue Sections

**Figure 4:**
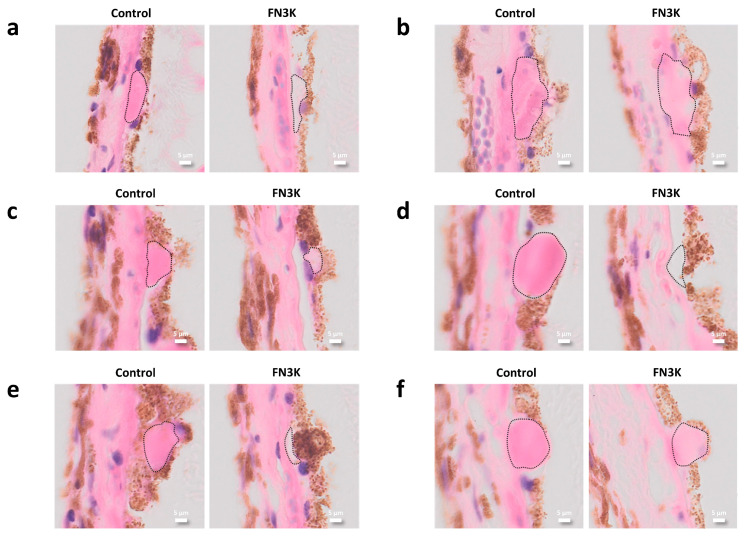
(**a**–**f**) Pairwise comparison on hematoxylin and eosin stained adjacent slides of 6 out of 17 control- and fructosamine-3-kinase (FN3K)-treated drusen originating from two patients with stage 3 AMD. Drusen are delineated with dotted lines. The scale bars are 5 µm.

Pairwise comparison of 17 drusen on adjacent human tissue sections revealed a visible color difference after FN3K treatment, as compared to control treatment with ATP/Mg^2^, which is suggestive of changes in their biochemical composition. A significant increase (*P* < 0.0001) in mean RGB color intensity (R + G + B/3) was found after FN3K treatment (205.8 ± 7.1) compared to control treatment (195.7 ± 7.3). Figure 4 illustrates the pairwise comparison of 6 out of 17 drusen after control and FN3K treatment. Microscopy revealed consistently several changes after therapy: less (Figure 4b,c,f) or absent (Figure 4a,d,e) drusenoid material, less well delineation (Figure 4c,f) and different staining intensity after FN3K treatment compared to control treatment. The HE staining of the drusen after control treatment was also more uniform than after FN3K treatment.

### 3.4. Near-Infrared Microspectroscopy on Stained Tissue Sections Unravels Biochemical Changes After FN3K Treatment

#### 3.4.1. Bruch’s Membrane

**Figure 5:**
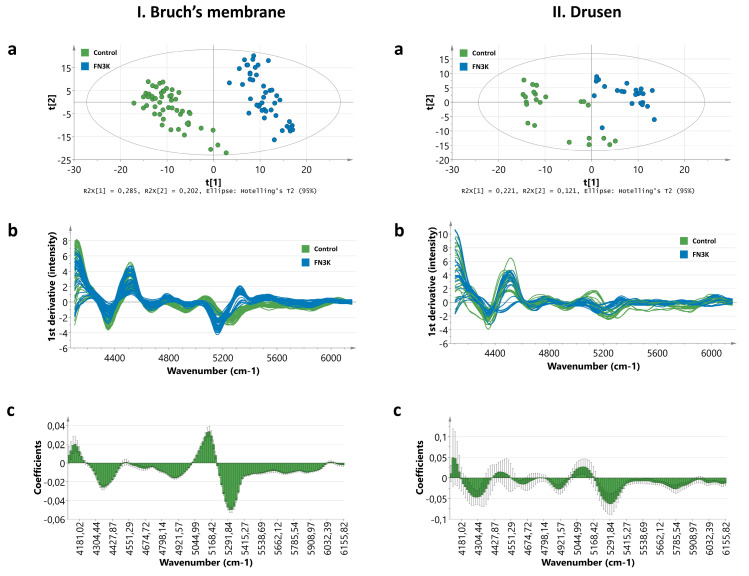
Near-infrared (NIR) microspectroscopy of the Bruch’s membrane (**I**) and drusen (**II**) on hematoxylin and eosin stained slides originating from two patients with stage 3 AMD. (**a**) Score plot showing clear clustering of the control-treated (green dots) and fructosamine-3-kinase (FN3K)-treated (blue dots) Bruch’s membrane and drusen based on principal component analysis (PCA) of the first derivative of the full spectral range (12,000 to 4000 cm^−1^). (**b**) Spectral differences in the first derivative of the 4000–6150 cm^−1^ range between the control-treated (green lines) and FN3K-treated (blue lines) Bruch’s membrane and drusen. (**c**) Coefficient plots resulting from partial least squares discriminant analysis (PLS-DA) of the 4000–6150 cm^−1^ range. The coefficients express how strongly the wavenumbers contribute in the discrimination between the control and FN3K-treated Bruch’s membrane and drusen.

Spectral data were extracted from a whole eye section originating from one patient with a considerable amount of drusenoid material and drusenoid lesions after ex vivo control (ATP/Mg^2+^) and FN3K treatment at 50 different locations in the Bruch’s membrane. PCA was performed on the first derivative of the full spectral range (SNV processed) and the resulting score plot showed clear clustering of the measurements performed on the control-treated (green dots) and the FN3K-treated section (blue dots, Figure 5Ia). Most spectral variation could be observed between 4000 and 6150 cm^−1^ (Figure 5Ib). Supervised PLS-DA analysis was performed on this region to reveal the most discriminative spectral changes. The resulting coefficient plot, which illustrates the change in the Y-variable (i.e., control versus FN3K treatment) when the X-variable (i.e., wavenumber) varies from 0 to 1 (in coded units while the other variables are kept at their averages), showed the highest regression coefficients around the peaks at 4350, 4921, 5245 and 5322 cm^−1^ (Figure 5Ic). The peak at 4350 cm^−1^, associated with O-H and C-O stretching combinations from glucose and CONH_2_ groups, showed an increased intensity after FN3K treatment. A clearly increased intensity after FN3K treatment was also observed around peak 4921 cm^−1^, a region attributed to the N-H/C-N combination band from primary amides (RCONH_2_). However, most expressed spectral variation could be observed in the region 5029–5400 cm^−1^. The peak at 5260 cm^−1^, associated with C=O carbonyl groups, showed a decreased intensity and remarkable shift towards 5160 cm^−1^ after FN3K treatment. In addition, a new peak appeared at 5322 cm^−1^.

#### 3.4.2. Drusen

Spectral data were acquired from ex vivo control-treated (*n* = 23) and FN3K-treated (*n* = 24) drusen originating from the eye sections of two patients with stage 2 AMD. PCA was executed on the first derivative of the full spectral range (SNV processed), and the resulting score plot showed a clear distinction between control- and FN3K-treated drusen (Figure 5IIa). The spectra contained considerable noise between 6500 and 12,000 cm^−1^. However, comparable to the measurements performed on the Bruch’s membrane, most spectral variation could be observed between 4000 and 6150 cm^−1^ (Figure 5IIb). The coefficient plot resulting from PLS-DA analysis on this region revealed the most prominent discriminative features in the regions around peaks 4350 and 5245 cm^−1^ (Figure 5IIc). While the peak at 4350 cm^−1^ also showed an increased intensity after FN3K treatment, the peak at 5245 cm^−1^ showed an increased intensity and shift towards 5168 cm^−1^. In addition, as observed for the Bruch’s membrane, a new, but more subtle, peak also appeared for the drusen around 5322 cm^−1^.

### 3.5. Ex Vivo Intravitreal FN3K Treatment on Human Eyes Strongly Reduces Size of Subretinal Drusenoid Deposits on Optical Coherence Tomography

**Figure 6:**
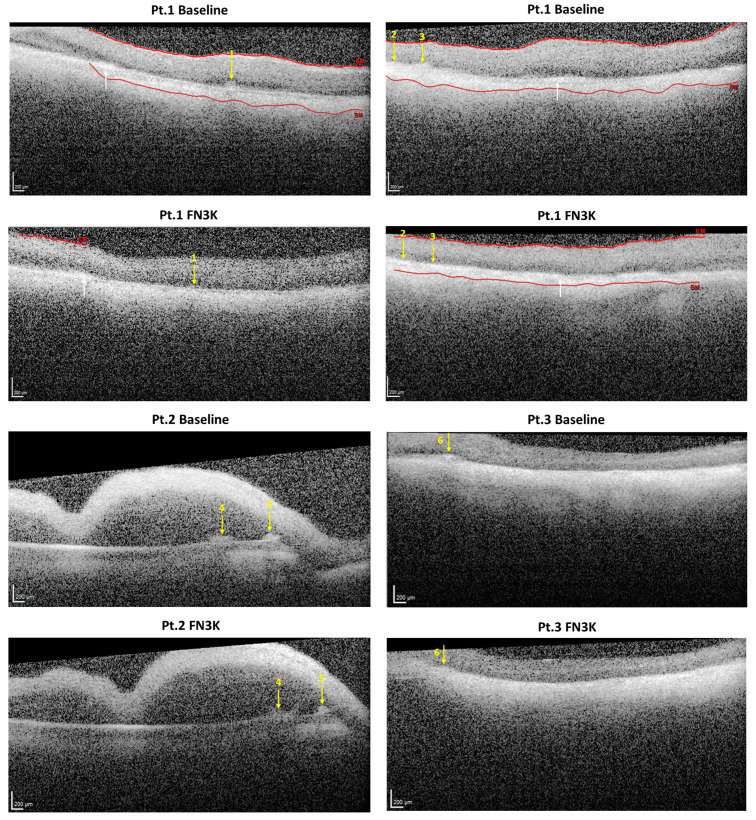
Optical coherence tomography (OCT) imaging of subretinal drusenoid lesions in post-mortem eyes of a 92-year-old patient with stage three AMD, i.e., right eye of Patient 1, a 74-year-old patient with stage 4 AMD (i.e., right eye of Patient 2) and a 79-year-old patient with stage 2 AMD (i.e., left eye of Patient 3) at baseline and 2 h after injection with a fructosamine-3-kinase (FN3K) solution. Subretinal drusenoid lesions (*n* = 6) are indicated with numbered yellow arrows. White arrows show highly intense zones, presumably corresponding to atrophic scar tissue. If recognized by the automatic detection system, the Bruch’s membrane (BM) and inner limiting membrane (ILM) are delineated with red lines. The scale bars are 200 µm.

**Figure 7:**
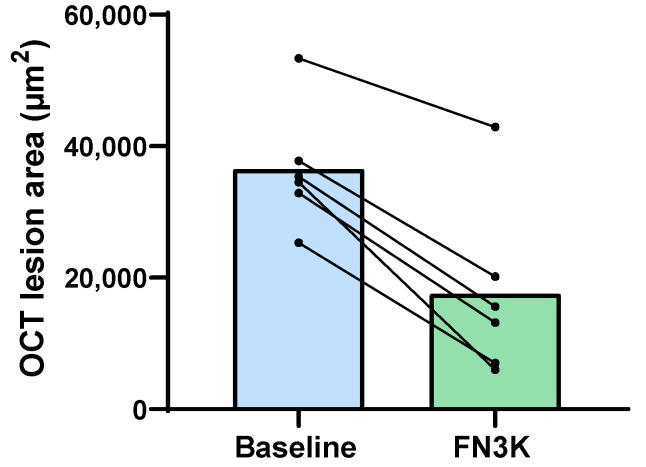
The area (µm^2^) of drusenoid lesions (*n* = 6) on optical coherence tomography (OCT) images in post-mortem human eyes from AMD patients (*n* = 3) at baseline and 2 h after injection with a fructosamine-3-kinase (FN3K) solution. The bars represent the mean.

In the right eye of the 92-year-old donor (i.e., Patient 1), three subretinal drusenoid lesions were successfully visualized. Only drusenoid deposits that disturbed the ellipsoid zone could be visualized clearly. After FN3K treatment, the drusen area decreased from 35,396 to 15,623 µm^2^ for the first druse (−56%), from 25,325 to 7039 µm^2^ for the second druse (−72%) and from 34,536 to 6011 µm^2^ for the third druse (−83%). In addition, two subretinal drusenoid lesions were detected in the right eye of the 74-year-old patient (i.e., Patient 2). The fourth lesion (area 53,355 µm^2^) disintegrated in two parts of 19,311 and 23,639 µm^2^, respectively (sum of both parts 42,949 µm^2^, −20%). The fifth lesion showed a decrease in its area from 32,879 to 13,193 µm^2^ (−60%). At last, one drusenoid lesion was visualized in the left eye of the 79-year-old patient (i.e., Patient 3). The sixth druse showed an area decrease from 37,794 to 20,185 µm^2^ (−47%). OCT images of the drusenoid lesions (indicated with numbered yellow arrows) at baseline and after FN3K treatment can be found in Figure 6. No changes could be observed in atrophic scar tissue after treatment (white arrows). Figure 7 illustrates the area decrease for each drusenoid lesion after FN3K treatment.

## 4. Discussion

In this study, we unraveled for the first time a potential role of recombinant FN3K and its cofactors in the treatment of AMD. Our study showed that both in vitro FN3K treatment of AGE-modified neural porcine retinas (i.e., photoreceptor cells) and ex vivo treatment of human neural retinas resulted in a significant decrease of autofluorescent AGEs. Furthermore, eyes treated intravitreally with FN3K showed no clear deposits of drusenoid material in contrast to their control-treated counterparts for both air- and cigarette-smoke-exposed mice. In addition, FN3K treatment of human whole eye sections resulted in reductions or complete disappearance of the areas and color intensities of drusenoid material beneath the RPE compared to control-treated sections. Differences in the biochemical composition of the Bruch’s membrane and drusen were confirmed using NIR microspectroscopy. Clear spectral differences were observed between control and FN3K-treated eye sections in the first derivative of the full spectral range. For both the Bruch’s membrane and the drusen, most spectral variation could be observed between 4000 and 6150 cm^−1^, a spectral region previously linked to glycation products [32]. The fact that biochemical differences could be observed in both drusenoid material in the Bruch’s membrane and drusenoid lesions suggests a potential role of our proposed treatment at different stages of the disease. At last, we showed that a single ex vivo intravitreal injection of FN3K was able to reduce (up to 83% decrease) the size of subretinal drusenoid deposits on OCT in post-mortem human eyes. However, it seems that, after a single intravitreal FN3K injection, only small drusenoid deposits dissolve after 2 h, while atrophic scar tissue remains unaffected.

Because AMD is a multifactorial and complex disease with an unknown etiology, there is a need for drugs that exert rather broad effects on retinal physiology [33]. While it is already well known that a FN3K-catalyzed pathway removes ketoamines and prevents AGE production [34], our findings indicate for the first time some potential of the enzyme in the disruption of retinal AGEs. Since AGEs are implicated in the pathogenesis of AMD [35], this could imply a possible role of FN3K as a readily available and innovative way to delay the progress of AMD and potentially reverse it. Recent findings show that proteins in drusen originate not only from the cellular debris of processed photoreceptors and RPE but also from the blood, suggesting that the process of drusen formation is at least partly comparable to the plaque formation in atheromatosis [25]. The accumulation of those glycated proteins into heavily crosslinked AGEs have been shown in Bruch’s membrane and drusen from ageing eyes and at increased levels in patients with AMD [5,6,36,37,38,39], which were significantly decreased after treatment with FN3K. Based on our results, FN3K seems to be a potential candidate to partially fulfill the need of preventing and reducing the amount of AGEs in the retina and Bruch’s membrane. Presumably AGEs also accumulate in RPE cells, where they can appear as either free adducts or AGE-modified proteins in lipofuscin granules [8]. A potential effect of FN3K on lipofuscin granules (autofluorescence at an excitation range of 450–490 nm) [40] cannot be excluded and warrants further research, as we only focused on Maillard-type autofluorescence.

The phenomenon of spontaneous and fast regression of drusen volume over time has been reported in several histopathological and clinical studies [41,42,43]. To date, the latter has been linked with dysregulations in the alternative pathway of the innate immunity [41,42]. However, based on our results, we could hypothesize that variations in endogenous ocular levels of FN3K might also be associated with fluctuations in the volume of drusen. As a comparison, it is already known that variations in FN3K levels may play a key role in the phenomenon of a discrepancy between glycated hemoglobin levels and other indicators of average glycemia (i.e., glycation gap) [34].

A drawback of this study was the limitation of treating subretinal drusenoid lesions ex vivo, as there are technical difficulties in detecting subRPE drusen in human post-mortem eyes. This can be overcome with an in vitro AMD model. However, it has to be mentioned that there is a scarcity of viable animal and cellular disease models [33,44]. Furthermore, developing reliable models is difficult since the initial events of AMD are caused by combinations of genetic and environmental factors that modify the photoreceptors, RPE, Bruch’s membrane and choriocapillaris, but not in a particular order [33,44,45]. In addition, while FN3K seems to be active against autofluorescent glycolaldehyde-derived AGEs, we currently do not know its potential effect on non-autofluorescent AGEs. While AGE antibody staining protocols for immunochemistry exist, their utility for our particular application is questionable since the exact targets of the commercial antibodies have not been determined so far. The latter is a restricting factor since AGEs are a heterogeneous group of compounds. Furthermore, the power of our study is limited by the small number of human donor eyes (nine donor eyes originating from seven different AMD patients). However, this is somehow compensated by the use of four different measurement techniques (i.e., fluorescence spectroscopy, light microscopy, NIR microspectroscopy and OCT imaging), all showing findings in line with our hypothesis, and the use of porcine and murine models. Moreover, due to the EU General Data Protection Regulation law on data protection and privacy, we were not able to retrieve information regarding donor carriage of any of the genes associated with AMD which may have an effect on the outcomes of this work.

It can be concluded that enzymatic treatment with FN3K forms a potential treatment option in the battle against AMD. While our preliminary research results need to be confirmed on larger sample sizes with improved phenotyping and genotyping, and by running human clinical trials, our findings open the door for future research. Since the exact AGE-related substrate of the enzyme is still unknown, future experiments should focus to unravel it. Moreover, diffusion experiments should be performed to investigate the ocular penetration of intravitreally injected FN3K and topical eye drops containing FN3K.

## 5. Patents

“Compositions for use to treat advanced glycation end product-dependent ocular diseases” patent filed at the European Patent Office. Priority date: 14 September 2018, international application number: PCT/EP2019/74058, international publication number: WO 2020/053188A1, international publication date: 19 March 2020.
